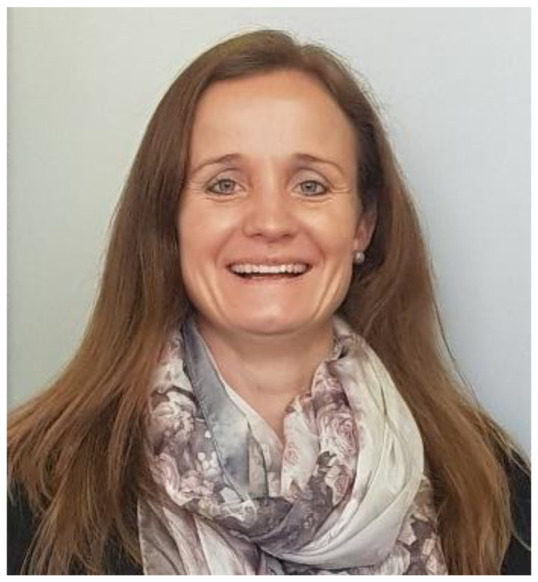# Partnerships between players, practitioners and researchers: the story of three stumps and two bails

**DOI:** 10.17159/2078-516X/2023/v35i1a15822

**Published:** 2023-06-05

**Authors:** Benita Olivier

**Affiliations:** Wits Cricket Research Hub for Science, Medicine and Rehabilitation, Department of Physiotherapy, School of Therapeutic Sciences, Faculty of Health Sciences, University of the Witwatersrand, Johannesburg, South Africa

In the 1700s, the wicket in cricket consisted of two stumps and a bail. Then, on 23 May 1775, during a match between Kent and Hambledon at the London Cricket Club, an incident occurred that changed the wicket forever. Lumpy Stevens bowled three deliveries at John Small, and the ball passed between the two stumps (with the bail remaining firmly intact) three times… consecutively.^[1]^ You can just imagine the outrage from the bowling side, being denied a wicket after beating the batting side thrice! This incident led to a law change where today the wicket consists of three stumps with two bails comfortably positioned in grooves on the top.

Interesting, hey?! Just like we need three stumps to form a wicket, we need three pillars for cricket success: the cricket player, the practitioner (such as coaches, sports scientists, biokineticists, physiotherapists, sports physicians, strength and conditioning trainers, sports coordinators, sports managers) and the researcher. These three pillars need to remain connected by the bails, i.e. good communication.

Researchers often formulate research questions based on recommendations from other researchers, as mentioned in published scientific papers. However, by opening the communication channels between players and their parents (in the case of minors), practitioners and researchers, we are bound to learn what the actual practical needs are. Research questions such as ‘what are the batting demands in women’s cricket?’, ‘how can we improve shoulder strength in cricket fast bowlers?’ and ‘how can we best manage lumbar bone stress injuries?’ are often born during conversations and discussions between players, practitioners and researchers.

Simultaneously, it is important for players and practitioners to engage in research, using Patient and Public Involvement (PPI) research designs, staying current with the latest findings, with the ultimate goal of applying research in the real world. Again, communication is essential between players, practitioners and researchers.

Players, practitioners and researchers need to form partnerships as described in our article titled ‘Injury surveillance in community cricket: a new innings for South Africa’ ^[2]^ and published as a State-of-the-Art feature in the South African Journal of Physiotherapy. This article describes how a partnership between a research entity and a cricket club or high school can impactfully enhance research. The research entity has needs and challenges but also concrete benefits to offer, while the same holds true for a cricket-playing high school or cricket club. Forming a partnership means the two entities get to develop a close relationship and work towards a common goal.

I know that this sounds easier said than done. While marrying a research entity and a cricket club is a great idea, it does require some high-level governance. In the meantime, we need to get going on the ground, or rather, ‘pitch’, where the action happens. How do we enhance communication between players, practitioners, and researchers? We need to be in the same room and join forces during conferences, journal clubs and case discussions. Author teams of academic papers need to consist of players, practitioners and researchers, and include a section where practical sense is made of the technical jargon.

This special issue in the South African Journal of Sports Medicine concerns ‘Cricket and aspects related to its Science, Medicine, and Rehabilitation’. It presents you, the reader, with a mix of valuable gems from the batting demands in Women’s Cricket; the kinematics of bowling and its relationship with ball release speed; the management of lumbar bone stress injuries; mental health in cricket, and the career development of South African cricketers, to mention a few.

This special issue is brought to you by the Wits Cricket Research Hub for Science, Medicine and Rehabilitation and was inspired by the 2022 Cricket Research and Practice Conference. The conference’s theme of ‘#gamechangingresearch’ speaks to the need for our research to be relevant and impactful, and translatable to clinical practice. Similar to how this conference brought together players, practitioners and researchers from around the world, this special issue aims to disseminate research from various subject areas, neatly bundled into one edition and tied together by the glue of cricket.

In cricket, the three stumps are connected by two bails. In the same way, we need to form partnerships to ensure communication between us, and ultimately use our research to inform practice and allow practice to inform our research. And although the aim of the game is to get the bails to fly, they are always placed back onto the stumps, before the game proceeds. Enjoy the read.[Fig f1-2078-516x-35-v35i1a15822]

**Figure f1-2078-516x-35-v35i1a15822:**